# Tumeurs de l'ovaire chez la femme ménopausée: à propos de 100 cas et revue de la literature

**DOI:** 10.11604/pamj.2014.19.235.4121

**Published:** 2014-10-31

**Authors:** Mehdi Kehila, Sahbi Kebaili, Samir Hidar, Sassi Boughizane

**Affiliations:** 1 Faculté de Médecine de Tunis, Service C du Centre de Maternité et de Néonatologie de Tunis, Tunie; 2 Faculté de Médecine de Sfax, Service de Gynécologie-Obstétrique de Sfax, Tunisie; 3 Faculté de Médecine de Sousse, Service de Gynécologie-Obstétrique de Sousse, Tunisie

**Keywords:** Kyste de l′ovaire, ménopause, diagnostic, traitement, Ovarian cyst, menopause, diagnosis, treatment

## Abstract

Le but de notre travail est d’étudier les particularités de prise en charge des tumeurs de l'ovaire chez la femme ménopausée. L’étude rétrospective porte sur 100 patientes opérées pour des tumeurs ovariennes en post ménopause durant une période de 5 ans. L’âge moyen des patientes était de 61,3 ans (extrêmes: 47- 84 ans). L'aspect échographique était liquidien pur dans 40% des cas, mixte ou solide dans 30% des cas. Le dosage de CA 125 était élevé dans 25% des cas. Un traitement chirurgical a été pratiqué chez toutes les patientes: Laparotomie de première intention dans 43 cas,cœliochirurgie dans 51 cas, cœlioscopie puis laparotomie dans 6 cas. L'examen anatomopathologique définitif a révélé 22% de tumeurs malignes et 10% de kystes fonctionnels. Le geste chirurgical était une annexectomie bilatérale pour la majorité des tumeurs bénignes et carcinologique en cas de tumeurs malignes. La stratégie diagnostique des tumeurs ovariennes en post ménopause reste de nos jours basée sur la clinique, l’échographie et les marqueurs tumoraux. Les bénéfices de la cœlioscopie sont indiscutables. L'attitude à opérer systématiquement les kystes uniloculaires ayant les critères de bénignité est actuellement révisée.

## Introduction

La découverte d'une masse annexielle en période post ménopausique est une situation fréquente et concerne 15 à 20% de cette population [1]. Le tiers de ces tumeurs sont malignes et ont pendant longtemps imposé la laparotomie exploratrice [1]. Toutefois, cette approche est responsable d'une morbidité thérapeutique importante chez des femmes âgées et le plus souvent tarées. Le développement de l’échographie gynécologique et les progrès techniques en imagerie médicale ainsi que les progrès de la coeliochirurgie ont modifié la prise en charge de ces tumeurs de l'ovaire.

## Méthodes

Nous nous sommes proposé d’étudier les particularités diagnostiques et de prise en charge des tumeurs de l'ovaire en post-ménopause. Pour cela nous avons réalisé une étude rétrospective sur 100 cas consécutifs de tumeurs ovariennes diagnostiquées à notre service chez des femmes en période de ménopause. Pour éviter les biais de sélection, nous sommes remontés à la période avant décembre 2003, date avant laquelle nous opérions de façon systématique tous ces kystes. Pour colliger les 100 cas consécutifs notre étude s'est étendue du 1er Avril 1999 au 31 décembre 2003 (soit 58 mois). Les cas ont été d'abord identifiés à partir des comptes rendu opératoires. Les données nécessaires à la réalisation de ce travail ont étés recueillis à partir des dossiers médicaux des patientes, des comptes-rendus opératoires et des comptes-rendus de l'examen anatomopathologique définitif. Les critéres d'inclusion étaient: une tumeur dont l'origine ovarienne a été confirmée lors de la chirurgie chez une patiente ménopausée depuis au moins un an et d’âge ≥ 50 ans. Les critères d'exclusion étaient: le stat ménopausique non précisé sur le dossier, une aménorrhée de moins de 1 an, un age < 50 ans et la présence d'une autre pathologie pouvant interférer avec la prise en charge thérapeutique (utérus polymyomateux, pathologie de l'endomètre, antécédent de deux laparotomies ou plus). Nous avons utilisé le logiciel statistique «SPSS version 10.0» pour l'analyse des données et l’étude statistique: le test de «Student» pour comparer les moyennes, le test de «chi-deux» et le test exact de «Fisher» pour comparer les pourcentages et le coefficient «Kappa» pour étudier la concordance entre les constatations opératoires et le résultat anatomopathologique final.

## Résultats

L’âge moyen des patientes était de 61,3 ans (extrêmes: 47-84 ans). Des antécédents médicochirurgicaux à type d'hypertension artérielle, de diabète, ou de cardiopathie ont été trouvés chez 43% des patientes. Concernant les antécédents de cancers: un antécédent de cancer du colon chez un parent de premiers degrés a été noté. Des antécédents personnels de cancer ont été trouvés chez 5 patientes (1 cancer de colon, 3 cancers du sein et 1 lymphome malin non Hodgkinien). Dix de nos patientes ont déjà eu une hystérectomie pour pathologie bénigne. Aucune patiente n'a été opérée pour un kyste de l'ovaire. Le motif de consultation était dominé par les douleurs pelviennes (64% des cas). Les autres signes (isolés ou associés) étaient des métrorragies (11 cas), une augmentation du volume de l'abdomen (17cas), une altération de l’état général (9 cas), des signes digestifs (4 cas) et urinaires (1 cas). A l'examen clinique, la tumeur n’était palpable que dans 54% des cas. Vingt patientes étaient asymptomatiques et la découverte était fortuite le plus souvent lors d'une échographie. L’échographie suspubienne et/ou endovaginale a permis d'objectiver la tumeur dans tous les cas. La taille tumorale moyenne était de 10,3 cm (extrêmes: 2,5 - 40 cm). L'aspect était liquidien pur dans 40% des cas et solide dans 8% des cas (Tableau 1). Le résultat du dosage du marqueur sérique CA 125 (demandé chez 76 patientes), n'a été mentionné que dans 24 dossiers. Le taux plasmatique de ce marqueur tumoral était inférieur à 35 UI/ml chez 18 patientes et supérieure à 35UI/ml chez 6 patientes. Aucune exploration doppler, ni imagerie par résonance magnétique de pelvis (IRM) n'a été pratiquée dans durant la période d’étude. Les 5 facteurs prédictifs de malignité qui ont été retrouvés dans notre étude étaient (Tableau 2): l'altération de l’état général (OR: 31,5; IC à 95% (3,7 - 269,6)); l'aspect mixte ou solide à l’échographie (OR: 11; IC à 95% (3,8-31,8)); la paroi épaissie à l’échographie. Le CA 125 > 35 (OR: 28; IC à 95% (2 - 293,4)); la présomption de malignité en per opératoire (OR: 60,7, IC à 95% (14,4 - 256,2)).

**Tableau 1:** Écho structure de la tumeur ovarienne

Echo structure	Fréquence	Pourcentage (%)
Anéchogène (Liquidienne pure)	40	40
Anéchogène + cloisons	14	14
Anéchogène + végétations intrakystiques	7	7
Hypoéchogène	9	9
Hétérogène (Mixte)	22	22
Echogène homogène (Solide)	8	8
Total	100	100

**Tableau 2:** Facteurs prédictifs de malignité

Facteurs prédictifs de malignité	Tumeurs malignes (%)	Tumeurs bénignes (%)	OR et IC à 95%	P
Altération de l’état général			31,5 (3,7-269,6)	<10-3
Oui (n = 9)	88,9%	11,1%		
Non (n = 91)	22,1%	77,9%		
Aspect mixte ou solide à l’échographie			11,0 (3,8-31,8)	<10-3
oui (n = 30)	60,7%	39,3%		
non (n = 70)	12,3%	87,7%		
Paroi épaisse à l’échographie			indéfini	<10-3

L'exploration à ciel ouvert ou par coelioscopie a conclu à une tumeur d'allure bénigne dans 69 cas et d'aspect malin dans 31 cas. L'examen extemporané a été réalisé chez 76 patientes. Il a conclu à une tumeur bénigne dans 50 cas, maligne dans 24 cas et borderline dans 2 cas. Le résultat de cet examen était concordant avec l'examen anatomopathologique définitif dans 100% des cas. Sur le plan thérapeutique, un traitement chirurgical a été pratiqué chez toutes les patientes (Tableau 3).

**Tableau 3:** Type d'intervention

Type d'intervention	Fréquence	Pourcentage
Coeliochirurgie	51	51%
Coelioscopie puis laparotomie	6	6%
Laparotomie de première intention	43	43%

Le geste chirurgical pour les tumeurs bénignes était: une annexectomie coelioscopique unilatérale dans 18 cas, bilatérale dans 27 cas, une annexectomie bilatérale à ciel ouvert dans 9 cas, annexectomie bilatérale avec hystérectomie pour une pathologie associée dans 15 cas. Pour les autres tumeurs, le traitement consistait en: annexectomie bilatérale avec hystérectomie pour les tumeurs borderlines, une chirurgie radicale première ou une chimiothérapie néoadjuvante pour le cancer de l'ovaire en fonction de stade de la maladie.

La répartition selon le type histologique à l'examen anatomopathologique définitif est résumée dans le (Tableau 4). Chez 3 patientes, le bilan clinique et radiologique initial était évocateur d'une tumeur maligne à un stade avancé et une coelioscopie première a été réalisée pour évaluer l'extirpabilité des lésions cancéreuses. Une biopsie tumorale et des gouttières pariétocoliques a été pratiquée dans 3 cas de tumeurs avancées avec carcinose péritonéale. L'extraction de la pièce opératoire a toujours été dans un sac endoscopique.

**Tableau 4:** Répartition selon le type histologique à l'examen anatomopathologique définitif

Type histologique	Fréquence	Pourcentage
Tumeurs bénignes	64	64%
Cystadénome séreux	34	34%
Cystadénome mucineux	12	12%
Kyste endometriosique	4	4%
Kyste dermoïde	3	3%
Fibrothécome	11	11%
Tumeurs malignes	24	24%
Cystadénocarcinome séreux	11	11%
Cystadénocarcinome mucineux	5	5%
Adénocarcinome papillaire séreux	4	4%
Carcinome peu différencié	2	2%
Carcinome endometrioïde	2	2%
Tumeurs borderlines	2	2%
Tumeur mucineuse borderline	2	2%
Kystes fonctionnels	10	10%

## Discussion

La découverte d'une masse annexielle en période post ménopausique est une situation fréquente qui concerne 15 à 20% de cette population [1]. La découverte d'une tumeur ovarienne à la ménopause a une valeur péjorative et fait craindre un cancer. Pour Koonings et al [2], le risque de malignité d'une tumeur de l'ovaire est multiplié par 12 si l'on compare les tranches d’âges 20-29 et 60-69. En plus, 80% des cancers de l'ovaire intéressent la femme de plus de 50 ans avec une fréquence maximale entre 50 et 59 ans [3]. Dans notre série, on a trouvé un taux de malignité de 26%. Ce taux peut atteindre 36,8% dans la littérature [4]. A la ménopause, un tiers des tumeurs ovariennes est donc un cancer. En cas de présomption de malignité, la prise en charge de ces tumeurs ne pose pas de problème et la laparotomie est à notre avis justifiée. De nombreux scores ont été proposés pour évaluer le risque de malignité d'une tumeur ovarienne. La plupart de ces modèles incluent le statut ménopausique, les anomalies échographiques et les dosages des marqueurs tumoraux avec une sensibilité qui atteint 80% [5]. Tingulstad et al. [6] ont montré que chez la femme ménopausée, le risque de malignité atteint 70% en cas d'association d'anomalies ovariennes échographiques (une image kystique hétérogène ou échogène, des végétations, la taille kystique > 8 cm) et d'un taux de CA125 > 22,2 UI/ml. Avec ces mêmes aspects échographiques, ce risque n'est atteint chez la femme non ménopausée que lorsque le taux de CA125 dépasse 66,7 UI/ml [6]. Pour Benacerraf et al. [7], l'association CA125 - échographie augmente la valeur prédictive négative de malignité. Au total, chez la femme ménopausée, lorsque l'examen clinique, l'aspect échographique et le CA 125 sont en faveur de la malignité, 75% des patientes auront la confirmation de la malignité lors de la chirurgie. Inversement, lorsque ces trois informations concordent vers la bénignité, moins de 5% des patientes ménopausées auront un cancer [8]. D'autres techniques d'imagerie médicale (Doppler pulsé, tomodensitométrie pelvienne (TDM), imagerie par résonance magnétique (IRM), PET Scan et plus récemment l’échographie Doppler 3D) ont été intégrées dans l'exploration préopératoire des tumeurs ovariennes à la ménopause [9]. Mais il n'existe pas d'examen qui permet un diagnostic de certitude [9]. La pertinence diagnostique de l’échographie endovaginale est supérieure à celle de la TDM. Le scanner a par contre un intérêt certain dans le cadre du bilan d'extension ou au cours de la surveillance post-opératoire. La pertinence diagnostique de l'IRM est légèrement supérieure à celle de la TDM [10, 11]. Ces deux techniques sont d'accès restreint et de coût non négligeable ce qui en limite la diffusion. Leurs indications doivent être envisagées de manière restrictive [10]. La scintigraphie au PET Scan présente également des performances intéressantes. Dans les premiers travaux publiés, on a observé une meilleure sensibilité et spécificité que l'IRM. Le PET Scan permet surtout de montrer une extension péritonéale ou épiploïque, non décelable sur les examens morphologiques habituels [11].

### Kyste d'allure fonctionnelle et ménopause

La difficulté de prise en charge se pose lorsqu'il s'agit d'un kyste ovarien simple chez une femme ménopausée (liquidien uniloculaire strictement anéchogène à paroi fine). Dans la littérature et dans des études qui ont consisté à réaliser une échographie transabdominale et endovaginale à des séries de femmes ménopausées asymptomatiques, sa prévalence varie entre 3,3 et 20,8% de l'ensemble de femmes ménopausées [12]. Dans notre série, il représentait à lui seul 40% de l'ensemble des tumeurs. Canis et al. sur 430 images kystiques pures étudiées par voie endovaginale ont trouvé 5 tumeurs borderline (1,2%) mais aucune tumeur maligne [13]. Dans notre série, 22,5% des kystes simples étaient des kystes fonctionnels (9 cas) et 77,5% des tumeurs bénignes (31 cas). Dans notre série, 45% des patientes avaient des antécédents pathologiques pouvant interférer avec le choix thérapeutique et il s'agissait d'une tare dans 30% des cas (cardiopathie ischémique, diabète mal équilibré ou hypertension artérielle mal équilibrée). La prise en charge d'un kyste uniloculaire anéchogène chez une femme ménopausée présente donc un dilemme: ne pas opérer abusivement des femmes le plus souvent tarées sans méconnaître un cancer de l'ovaire à un stade précoce. La taille du kyste et le dosage du CA 125 permettent-il d’éviter ces deux pièges? Plusieurs études ont montré que le risque de malignité de kystes uniloculaires chez la femme ménopausée est faible et est corrélé à la taille kystique. Lorsque la taille kystique est < 5 cm, la malignité chez la femme ménopausée est très rare [1, 12, 14]. En outre, plus que 50% de kystes simples disparaissent spontanément au bout de 3 mois. Il est donc raisonnable de proposer une attitude conservatrice en cas de kyste simple avec une surveillance échographique et d'intervenir en cas d'apparition de symptômes ou de modification échographique [15]. Une étude internationale multicentrique réalisée en 1998 a montré que les femmes ménopausées avec un kyste ovarien asymptomatique de moins de 5 cm de diamètre et un taux de CA 125 normal ont un risque nul de cancer [16]. Par contre, une tumeur ovarienne chez une femme ménopausée avec une ascension progressive du CA 125 est presque toujours une tumeur maligne [17].

### Cœlioscopie et diagnostic de malignité

La coelioscopie peut être considérée comme une étape diagnostique supplémentaire. Cette étape est très importante vu que le bilan préopératoire n'est pas fiable à 100% [18]. La fiabilité de la coelioscopie pour le diagnostic de malignité ou de bénignité a été étudiée par plusieurs auteurs. Canis et al. [19] dans une étude rétrospective incluant 1098 patientes ont évalué la concordance entre le diagnostic macroscopique chirurgical et le diagnostic histologique définitif. Ils ont montré que l'exploration coelioscopique est sûre et fiable avec une sensibilité de 100%, une spécificité de 93,5%, une VPP de 37,8%, et une VPN de 100%. Dans notre série 57 coelioscopies ont été effectuées. La concordance avec l'examen anatomopathologique définitif concernant le diagnostic de bénignité et de malignité était de 100%.

### Cœliochirurgie en cas de présomption de bénignité

Après avoir exclu les principaux signes de malignité (végétations intra ou extra-kystiques, tumeurs de couleur inhabituelle, vascularisation abondante et anarchique), le traitement coeliochirurgical peut être réalisé. L'annexectomie bilatérale est le geste classiquement réalisé chez une femme ménopausée présentant une tumeur ovarienne. Cette technique s'applique à toute pathologie kystique ovarienne où le diagnostic de bénignité est une quasi-certitude. Elle est de réalisation rapide et entraîne des suites opératoires simples. Dans certains cas, lorsque l'annexe sain est d'abord chirurgical difficile avec un risque de plaies digestives, vasculaires ou vésicales, l'annexectomie unilatérale se justifie. Les principales complications spécifiques de cette chirurgie sont: l'essaimage ou la dissémination de cellules néoplasiques au niveau péritonéal en cas de tumeur maligne méconnue rompue et la récidive néoplasique au niveau des trous des trocarts [20]. La prévention du risque d'essaimage repose sur la bonne sélection des patientes et la prévention de contamination des trocarts repose surtout sur l'utilisation de sacs d'extraction. Le diagnostic de malignité à l'examen extemporané doit toujours imposer outre la conversion en laparotomie médiane, la reprise chirurgicale de tous les trous de trocarts, emportant une large collerette de sécurité (peau, graisse, aponévrose, muscle, péritoine). La laparotomie ne doit pas être considérée comme un échec, mais comme une solution. Il est important pour le chirurgien d’évaluer la difficulté technique pour proposer la chirurgie conventionnelle de première intention ou de prévenir la patiente d'une conversion possible.

L'utilisation de la coelioscopie dans la stadification de la tumeur ovarienne et dans l’évaluation de l'extirpabilité des lésions cancéreuses n'est pas clairement définie. Vergote et al [21] étaient les premiers à rapporter l'utilisation de cette voie d'abord en cas de tumeurs malignes de l'ovaire. Leur étude a montré qu'il n'y a pas une différence significative en terme de survie entre abord coelioscopique et laparotomie. Dans l’étude de Fagotti et al [22] portant sur 64 patientes porteuses de cancers ovariens avancés, la coelioscopie a permis une évaluation de la résécabilité tumorale comparable à la laparotomie. Aucun cas jugé non résécable par la coelioscopie n'a été jugé différemment par la laparotomie (VPN = 100%). Pomel et al [23], ont aussi conclu que la coelioscopie est une technique fiable pour explorer la carcinose péritonéale et évaluer l'extirpabilité des lésions cancéreuses chez les femmes porteuses des tumeurs ovariennes avancées.

## Conclusion

Les tumeurs ovariennes chez la femme ménopausée représentent une pathologie fréquente et la prise en charge doit toujours être prudente et attentive du fait de la crainte de la malignité. L’étape diagnostique initiale reste basée sur la triade: clinique, échographie et marqueurs tumoraux. En se basant sur les données de la littérature, l'attitude à surveiller les kystes uniloculaires asymptomatiques ayants les critères de bénignités est justifiée vue le risque infime de malignité rajouté aux risques opératoires en post ménopause. Les bénéfices de la coelioscopie sont indiscutables sous réserve d'un respect absolu des règles carcinologiques.
